# A randomised study in young subjects of the effects of eating extra fruit or nuts on periodontal inflammation

**DOI:** 10.1038/bdjopen.2017.22

**Published:** 2018-01-05

**Authors:** Sara Fridell, Edvin Ström, Christian Agebratt, Per Leanderson, Hans Guldbrand, Fredrik H Nystrom

**Affiliations:** 1 Centre for Oral Rehabilitation, Public Dental Health Care, County Council of Östergötland, Linköping, Sweden; 2 Department of Medical and Health Sciences, Faculty of Medicine and Health Sciences, Linköping University, Linköping, Sweden; 3 Department of Clinical and Experimental Medicine. Faculty of Medicine and Health Sciences, Linköping University, Linköping, Sweden

## Abstract

**Objectives/Aims:**

Fruit is often advocated as a healthy source of nutrients and vitamins. However, the high contents of sugars in many fruits could potentially counteract positive effects on the teeth.

**Materials and methods:**

We recruited 30 healthy non-obese participants who were randomised to either supplement their diet with extra fruits or nuts, each at +7 kcal/kg body weight/day, for 2 months.

**Results:**

Fructose intake increased from 9.1±6.0 to 25.6±9.6 g/day, *P*<0.0001, in the fruit group and was reduced from 12.4±5.7 to 6.5±5.3 g/day, *P*=0.007, in the nut group. Serum-vitamin C increased in both groups (fruit: *P*=0.017; nuts: *P*=0.009). α-Tocopherol/cholesterol ratio increased in the fruit group (*P*=0.0033) while β-carotene/cholesterol decreased in the nut group (*P*<0.0001). The amount of subjects with probing pocket depths ⩾4 mm in the fruit group was reduced (*P*=0.045) according to blinded examinations, and the difference in the changes in probing pockets ⩾4 mm was also statistically significant between the food groups (*P*=0.010).

**Conclusion:**

A large increase of fruit intake, compared with nuts, had a favourable effect on periodontal status in some respects, despite the high sugar contents. To search for potential protective micronutrients in fruit that protect the teeth could be an aim for further research.

## Introduction

Consumption of diets that are rich in vegetables and vitamin C appears to associate positively with periodontal health.^1^ Conversely, periodontal disease can progress rapidly in populations with undernourishment.^1^ Case–control studies show that patients with periodontitis exhibit low serum antioxidant micronutrient levels.^2–4^ Indeed, an encapsulated fruit, vegetable and berry juice concentrate was shown to improve gingival condition when this was added to non-surgical professional dental care in a randomised trial.^5^ Presence of periodontal disease is associated with signs of systemic low-grade inflammation,^6^ and also with the incidence of cardiovascular events.^7^ Gingivitis is a reaction on dental plaques that might affect as many as 15% of adults in Sweden.^8^ The standard method to diagnose gingivitis is to determine the frequency of bleeding on probing (BOP).

A high intake of fruit is often advocated as a nutrient source, which is rich in vitamins, antioxidants and fibre, and this is supposed to counteract both inflammation and cardiovascular disease. Although antioxidants and other micronutrients in fruits could potentially act to reduce periodontal disease, and the accompanying inflammation, fruits also often contain large amounts of sugars that could lead to increased gingival bacterial growth. Indeed, beneficial changes in the oral microbiota were reported in humans who were put on a ‘Stone Age’ diet for 4 weeks in which there was no access to refined sugar.^9^ Some fruits are acidic and this could be hazardous for the enamel. The American Dental Association lists citrus fruit as one out of the nine top foods that ‘damage your teeth’.^10^

We performed a randomised dietary trial in healthy participants with a duration of 2 months. The participants were asked to either supplement the diet with fruits or with nuts, each at +7 kcal/kg body weight/day. Serum levels of carotenoids and vitamins C and E were assessed to gain information about uptake of these micronutrients when the food composition was changed, and these results have been published earlier.^11^ The aim of the analyses from the study presented herein was to determine the effects of a large increase in the intake of fruits or nuts on probing pocket depths, levels of vitamins in serum and inflammation in non-obese healthy individuals.

## Materials and methods

### Subjects

The participants were recruited through local advertising in the area of Linköping University in south-east Sweden. Significant pre-existing medical conditions, including use of thyroid hormone replacement therapy or oral cortisone were exclusion criteria. Ongoing dietary supplementation by vitamins were not allowed during the trial.

### Laboratory investigations

Blood was drawn in the morning after a 10 h overnight fast. Plasma levels of the carotenoids: lutein+zeaxanthin; β-cryptoxanthin; lycopene; α- and β-carotene; and vitamin E were determined by high-performance liquid chromatography as described earlier.^12^ Levels of vitamin C were analysed with high-performance liquid chromatography as has been described,^13^ however, a gradient elution starting with a mobile phase consisting of 50 mmol/l KH_2_PO_4_, pH 3.2 and ending with 100% acetonitrile was added to obtain a more efficient washing of the column between injections. The total coefficient of variation was 8.5%.

Analysis of lipopolysaccharide (*Escherichia coli*, L-2654, Lot-ID (Sigma-Aldrich, St Louis, MO, USA))-stimulated interleukin-1β (IL-1β), interleukin-6 (IL-6) and tumour necrosis factor α was performed by Duplex Luminex 200 (Invitrogen, Stockholm, Sweden) according to the instructions by the manufacturer (Magnetic Luminex Performance Assay Human Base lit A, R&D Systems, Minneapolis, MN, USA).

### Dental examination

The dental examination included registration of periodontal parameters, dental caries and dental erosions. One previously calibrated dentist, a postgraduate student (SF) at Centre for Oral Rehabilitation, Linköping, Sweden a specialist clinic for periodontology, performed all the examinations. All participants were examined at baseline and at the end of the study, and the dental examiner was blinded with regard to results of the randomisation. The periodontal examination involved assessment of the plaque index.^14^ Probing pocket depths were recorded on six surfaces (mesio-buccal, mid-buccal, distobuccal, mesio-lingual, mid-lingual and disto-lingual) of each tooth using the manual periodontal probe PCP 11 (Hu-Friedy, Chicago, IL, USA). The pocket depths were recorded if they were 4 mm or deeper. BOP was recorded after probing of the pockets. The percentages of total number of sites that bled were recorded for each participant. Clinical visible caries and dental erosions were registered. The buffer capacity was measured with Dentobuff Strip (Orion Diagnostica, Espoo, Finland). All subjects filled in a questionnaire to determine potential changes in oral hygiene routines.

### Nutritional intake

The participants were provided with scales (Soehnle 66100, Nassau, Germany) and notebooks to weigh and record food items that were consumed during 3 consecutive days before and at the end of the study.^11^

### Intervention

The participants were randomised, by drawing ballots, to eat an additional 7 kcal/kg body weight/day of extra fruit or nuts, after the baseline investigations had been performed. The randomisation ratio was 1:1. For example, this means that a subject weighing 65 kg was asked to consume a daily extra intake of ~7 apples or 70 g of walnuts. The study lasted 8 weeks for each individual and the participants bought and consumed the fruits or nuts individually and kept diaries of consumption throughout the study. Subjects were consecutively reimbursed for the extra cost of the fruit or nuts based on the information from collected receipts. The study ran from September to December 2014.

### Ethics

The study was approved by the Regional Ethics Committee of Linköping and performed in accordance with the Declaration of Helsinki of 1975. The ClinicalTrials.gov registration number was NCT02227511.

### Statistical analyses and power calculation

Statistical estimates were calculated using IBM SPSS Statistics 23 software (IBM Corporation, Somers, NY, USA). Comparisons within and between groups were done with Student’s paired and unpaired two-tailed *t*-test for normally distributed data or as stated in the Results section. Mean values and s.d.’s are given. Statistical significance refers to two-sided *P*⩽0.05. Levels of vitamin C that could not be detected in plasma were set to 1 μmol/l in statistical calculations as the lower limit of detection for vitamin C was 2 μmol/l. On the basis of our earlier intervention studies of hepatic fat content ^15,16^ the study had 80% power to detect a 50% change in hepatic fat content within either group. No formal power calculation was carried out with regard to effects on dental status, as we had no access to any data to carry out such analyses. We could not find any published investigations with similar aims and measures as the one presented herein, and in this sense, this was a pilot study.

## Results

Forty potential participants responded to the advertising and the first 30 of these were consecutively enrolled in the study. None of these had any of the exclusion criteria. The participants consisted of 18 males and 12 females (7 men and 8 women in the fruit group, and 11 men and 4 women in the nut group) with a mean age of 23.5±3.7 years and a mean body mass index of 22.3±1.9 kg/m^2^. Twenty-nine participants were students at Linköping University, and one was a university employee. According to questionnaires there was one regular smoker in the nut group and none in the fruit group. Snuff use was not part of the standard questions but when asked in retrospect, one participant in the fruit group and one more in the nut group were regular users of snuff. There were no dropouts during the trial. Table 1 shows anthropometric data. All participants except two reported that they brushed their teeth two times a day and the remaining two participants reported brushing their teeth once a day. The frequency of brushing teeth did not change in any of the participants during the study.

The intake of fructose increased in the fruit group and was reduced in the nut group (*P*<0.0001 for change between groups; Table 1) according to the diet registrations. When the intakes from different kind of nuts were compared and expressed as g/day, the most common sources were from cashews (47.4%), peanuts (14.1%), walnuts (8.1%), almonds (8.0%), pistachios (7.8%), hazelnuts (5.8%) and Brazil nuts (3.5%) with <1% coming from other specified nuts. A total of 3.4% of the nuts was from nut mixes. The corresponding proportions of different fruits consumed in the fruit group were bananas (38.7%), apples (19.4%) citrus fruits (14.8%), pears (8.2%), melons (3.9%), grapes (3.2%), mangos (3.0%), kiwis (2.2%) and persimmons (1.8%) with <1% each from other specified fruits. The compliance expressed as reported intake of nuts or fruit in kcal/day/kg body weight during the study period, compared with the goal of 7 kcal/day/kg body weight, was 107.6±8.8% in the nut group and 98.8±9.4% in the fruit group.

Table 2 shows effects of the interventions on vitamin levels in plasma. Levels of vitamin C was lower in the nut than in the fruit group at baseline and at the end of the trial but the increase was numerically similar in the two groups (Table 2). The α-tocopherol/cholesterol ratio increased in the fruit group and decreased numerically in the nut group, the difference in changes between groups was statistically significant (Table 2; *P*=0.005). The ratio β-carotene/cholesterol decreased in the nut group (*P*<0.0001), as seen in Table 2.^11^

The effects on dental status by the interventions are shown in Table 3. In the fruit group, there was a decrease in the total number of probing pockets with a depth of 4 mm or more whereas there was a trend towards an increase of this parameter in subjects randomised to consuming more nuts as seen in Table 3. The difference in the changes of the total amounts of probing pocket depths of the participants being at least 4 mm deep was statistically significant when compared between groups (Table 3; *P*=0.010). The plaque indexes and fraction amount of teeth BOP were unchanged in both groups. As shown in Table 4 there was no changes in levels of high-sensitive CRP in the study, and levels were generally low. Lipopolysaccharide-stimulated production of IL-1β by white blood cells decreased in the nut group (Table 4). Serum IL-6 was detectable in five subjects at baseline and only one subject had procalcitonin levels above detection limit at start of the study. After 8 weeks none had detectable levels of procalcitonin while there were still five individuals who had detectable serum IL-6 levels. Thus, the serum levels of IL-6 and procalcitonin were unaffected by consuming either extra nuts (paired *t*-test for IL-6: *P*=0.29; and for procalcitonin: *P*=0.33) or fruits (paired *t*-test for IL-6: *P*=0.30; and for procalcitonin: *P*=1).

We did a *post hoc* analysis of potential impact of the acidity of the fruit that had been consumed according to the diaries. An intake of fruits with high acidity (pH<4.05 (^ref. 17^)) constituting <10% of the total fruit was found in 9 of the 15 participants. Within the ‘low-acidity’ subgroup the teeth with probing pocket depths of 4 mm or more decreased from 1.11±1.2 to 0.444±0.88, *P*=0.50, while plaque indexes and BOP did not change significantly (*P*=0.71 and 0.85, respectively). In the contrasting group reporting to have chosen fruits with 10% or more with a high acid content we found that teeth with probing pocket depths of 4 mm or more were numerically reduced from 1.33±1.5 to 0.667±0.82, *P*=0.36 while plaque index (*P*=0.81) and BOP (*P*=0.92) were unchanged. There were 4 subjects in the fruit group who changed behaviour and reported that they performed interproximal dental care every day at the end of the trial compared to every other day at baseline. We tested whether the reported frequency of interproximal dental care was inter-related to any dental outcomes, but found no significant impacts on whether this was performed at least every other day or less frequently. The change in probing pocket depths of 4 mm or more between the fruit and nut groups remained statistically significant also after removal of the 4 participants who reported more frequent interproximal dental care at end of the study (*P*=0.038). Also, exclusion of the participant who was a smoker from the analysis of change in probing pocket depths of 4 mm or more did not affect the statistical significance between groups (*P*=0.006).

## Discussion

We designed this trial to compare increased consumption on food based on carbohydrates, i.e., fruit, with iso-caloric food sources that were instead high in fat and protein, in the nut group. In contrasts to our expectations, we found reduced number of probing pocket depths of 4 mm or more in the fruit group while there was a trend to an increase of such pockets in the nut group. This was not caused by changes in the habits to brush teeth in any of the groups, according to data from questionnaires. Although four participants reported an increase in interproximal dental care in the fruit group, removal of these subjects from the analyses did not change the statistical significance of the changes between the groups. However, as the number of sites with probing pocket depths ⩾4 mm were low, our findings should be interpreted with due caution. Gingivitis is a reversible condition and probing pocket depths reflects the situation at the specific examination day.

There were increases in plasma levels of vitamin C in both groups, making it unlikely that vitamin C was linked with the differing changes in probing pocket depths. The systemic inflammation measured as high-sensitive CRP was low in both group throughout the trial. However, lipopolysaccharide-stimulated IL-1β from isolated leucocytes were reduced in the nut group, which was thus a finding in contrast to the trend for an increase in the number of probing pocket ⩾4 mm. Thus, our study does not support that the change in probing pocket depths was related to systemic inflammation in this cohort of non-obese healthy young subjects. Our *post hoc* analysis of impact of the level of acidity in the chosen fruit displayed similar trends in subjects that had chosen fruits with different acidities. The frequent claim that fruit, in particular if it is acidic, can harm teeth has not based on randomised trials, according to published data that we scrutinised, and the trial presented here does not support such warnings. If there were firm data behind the warning from American Dental Association to have citrus fruit as a top food that can ‘damage your teeth’,^10^ we would have expected to find a statistical trend to support importance of acidity of the fruit in our study.

We could not detect changes in plasma levels of any vitamins or other micronutrients that reflected the effects on dental health in any of the groups. However, there are numerous more nutrients and antioxidants that could be tested in future trials on this topic. However, any protective component in the fruits would likely have to be common in the fruits as our design allowed participants to freely choose the fruits and were also allowed to consume berries. We call for further randomised studies on this important health topic in order to collect a firm basis for dietary recommendations aimed at achieving good dental health in order to diminish inflammatory harm from sugars.

To the best of our knowledge, only one prospective randomised controlled trial of the effects of nutritional supplements upon periodontal outcomes of reasonable duration has been published. This trial was conducted by Chapple *et al.*^5^ and it was based on a cohort of patients with chronic periodontitis. The subjects in the trial by Chapple *et al.* were randomly assigned to one of three groups to be given capsules containing concentrates of either of the following: fruits and vegetables; fruits, vegetables and berries; or placebo. These capsules were provided in addition to non-surgical periodontal therapy for all participants. Chapple *et al.*^5^ found that the combination of fruit and vegetable concentrate, in particular, reduced pocket depth as compared with placebo after 2 months. Our findings are in agreement with the outcome of the study by Chapple *et al.* and both studies imply protection from micronutrients on dental health. However, as a consequence by using capsules, the study by Chapple *et al.* precluded an additional local effect from acids or sugars from the nutrients, as was part of our trial. Chapple *et al.*^5^ did not report effects on inflammatory markers.

The reduction of β-carotene in the nut group is in line with the small amounts of β-carotene that is usually present in nuts. The small but significant increase in α-tocopherol among participants in the fruit group was somewhat unexpected as tocopherols are more associated with the consumption of nuts and seeds^18^ than with fruit intake. However, to some extent tocopherols can also be found in fruits and the participants did indeed eat large amount of fruits in this trial.

Limitations of the study include the duration of 2 months and that we only included healthy non-obese individuals who were young. It could also be argued that it was a drawback that no specific kind of fruits or nuts were advocated, or even provided, to the participants. The small size of study is also acknowledged as a limitation.

In summary, the results of this randomised trial did not show that consuming large amounts of fruits had negative effects on periodontal health or inflammation during 2 months. In contrast, we found improvements with regard to the amount of probing pocket depths by consuming extra fruit as compared with the extra nuts. However, there were no detectable changes in measures of systemic inflammation in this group of non-obese young participants that corresponded to changes in periodontal health. It would be of great interest to further find if there are specific micronutrients in fruits that counteract the gingivitis and inflammation that is supposedly induced by the high sugar contents of many fruits.

## Figures and Tables

**Table 1:** Baseline data and effects of the interventions

*Variable*	*Group*	*Baseline*	*After 8 weeks*	P *within groups*	P *for difference of changes between groups*
Weight (kg)	Fruit	66.45±8.70	67.15±9.04	0.13	0.95
	Nut	73.61±9.01	74.28±9.02	0.049	
BMI (kg/m^2^)	Fruit	22.15±1.61	22.30±1.7	0.24	0.83
	Nut	22.54±2.26	22.73±2.28	0.045	
Energy intake (kcal/day)	Fruit	2635±933	2663±773	0.9	0.37
	Nut	2519±721	2763±595	0.035	
Fructose intake (gram/day)	Fruit	9.1±6.0	25.6±9.6	<0.0001	<0.0001
	Nut	12.4±5.7	6.5±5.3	0.007	
Triglycerides (mmol/l)	Fruit	0.80±0.39	0.84±0.38	0.57	0.20
	Nut	0.88±0.36	0.80±0.33	0.20	
Cholesterol (mmol/l)	Fruit	4.44±0.88	4.38±0.91	0.49	0.46
	Nut	4.09±0.52	3.95±0.45	0.09	
HDL cholesterol (mmol/l)	Fruit	1.57±0.36	1.55±0.43	0.41	0.75
	Nut	1.40±0.26	1.39±0.22	0.83	
LDL cholesterol (mmol/l)	Fruit	2.54±0.75	2.43±0.69	0.13	0.85
	Nut	2.33±0.55	2.20±0.42	0.11	
HbA1c (mmol/mol)	Fruit	32.5±2.4	31.8±2.2	0.028	0.51
	Nut	31.9±3.3	31.6±3.7	0.68	

Abbreviations: BMI, body mass index; HbA1c, glycated haemoglobin; HDL, high-density lipoprotein; LDL, low-density lipoprotein

**Table 2:** Levels of plasma vitamin C (μmol/l) and the ratios of vitamin E and carotenoids per mmol/l of cholesterol at start and after 8 weeks

*Variable*	*Group*	*Levels at baseline*	*Levels after 2 months*	P *for change within group*	P *for difference of changes between groups*
P-vitamin C (μmol/l)	Fruit	16.3±11^a^	28.7±15^a^	0.017	0.53
	Nut	8.7±6.0^a^	17.2±9.4^a^	0.009	
α-Tocopherol (μmol/l/mmol/l cholesterol)	Fruit	6.1±0.71	6.65±1.4	0.033	0.005
	Nut	6.6±0.64	6.24±0.71	0.067	
γ-Tocopherol (μmol/l/mmol/l cholesterol)	Fruit	0.34±0.14	0.35±0.11	0.82	0.13
	Nut	0.37±0.17	0.47±0.15	0.081	
α-Carotene (μmol/l/mmol/l cholesterol)	Fruit	0.087±0.048	0.078±0.032	0.29	0.75
	Nut	0.076±0.038	0.063±0.047	0.083	
β-Carotene (μmol/l/mmol/l cholesterol)	Fruit	0.25±0.10	0.23±0.12	0.40	0.26
	Nut	0.25±0.079	0.21±0.059	<0.0001	

Data are means±s.d.

Abbreviation: P, plasma.

a The differences in levels of vitamin C levels were statistically significant between groups at baseline (*P*=0.023) and at the end (*P*=0.017) of the trial.

**Table 3:** Dental status before and after the interventions in the two groups

*Variable*	*Group*	*Baseline*	*After 8 weeks*	P *within groups*	P *for difference of changes between groups*
Plaque index (%)	Fruit	27.3±20	26.9±21	0.92	0.79
	Nut	20.7±16	18.6±17	0.63	
Bleeding on probing (%)	Fruit	18.9±16	18.3±13	0.83	0.81
	Nut	12.2±14	10.5±8.6	0.57	
Probing pockets ⩾4 mm (*n*)	Fruit	1.2±1.3	0.53±0.83	0.045	0.010
	Nut	0.53±0.83	1.3±1.5	0.094	

**Table 4:** Effects of the interventions on markers of inflammation

*Variable*	*Group*	*Baseline*	*After 8 weeks*	P *within groups*	P *for difference of changes between groups*
HsCRP (mg/l)	Fruit	1.15±1.5	0.99±1.0	0.38	0.41
	Nut	0.61±0.66	0.76±1.1	0.67	
IL-6 (pg/ml)	Fruit	26.4±10	28.7±9.7	0.31	0.26
	Nut	28.4±15	26.6±12	0.27	
IL-1β (pg/ml)	Fruit	9.77±6.9	9.47±7.0	0.86	0.16
	Nut	8.97±5.5	7.34±4.1	0.030	
TNFα (pg/ml)	Fruit	6.84±2.6	7.65±2.0	0.16	0.41
	Nut	6.77±2.9	6.93±2.0	0.72	

Levels of IL-6, IL-Iβ and TNFa were determined in white blood cells by CPR after stimulation of blood samples for 1 h with LPS.

Abbreviations: HsCRP, high-sensitive CRP; IL, interleukin; LPS, lipopolysaccharide; TNFα, tumour necrosis factor α.
